# Machine learning for identifying liver and pancreas cancers through comprehensive serum glycopeptide spectra analysis: a case‐control study

**DOI:** 10.1002/1878-0261.70084

**Published:** 2025-06-30

**Authors:** Motoyuki Kohjima, Yuko Takami, Ken Kawabe, Kazuhiro Tanabe, Chihiro Hayashi, Mikio Mikami, Tetsuya Kusumoto

**Affiliations:** ^1^ Department of Gastroenterology NHO Kyushu Medical Center Fukuoka Japan; ^2^ Department of Hepato‐Biliary‐Pancreatic Surgery NHO Kyushu Medical Center Fukuoka Japan; ^3^ Medical Solution Promotion Department Medical Solution Segment, LSI Medience Corporation Tokyo Japan; ^4^ Chigasaki Central Hospital Women's Center Kanagawa Japan; ^5^ Department of Gastrointestinal Surgery and Clinical Research Institute Cancer, Research Division NHO Kyushu Medical Center Fukuoka Japan

**Keywords:** blood‐based diagnostics, glycopeptides, liver cancer, machine learning, pancreatic cancer, tumor markers

## Abstract

Liver and pancreatic cancers are difficult to detect early, leading to high mortality rates. Blood‐based diagnostics present a viable alternative for earlier detection, potentially improving survival rates. The c*omprehensive serum glycopeptide spectra analysis* (CSGSA) method combines enriched glycopeptides (EGPs) with conventional tumor markers through machine learning to accurately identify early stage cancers. Here, we analyzed nine tumor markers (CA19‐9, AFP, PSA, CEA, CA125, CYFRA, CA15‐3, SCC antigen, and NCC‐ST439) in 119 patients with pancreatic cancer and 49 with hepatocellular carcinoma, alongside 590 healthy controls. We also analyzed EGPs using liquid chromatography‐mass spectrometry. We found that α1‐antitrypsin with a fully sialylated biantennary glycan at asparagine 271 and α2‐macroglobulin with a fully sialylated biantennary glycan at asparagine 70 effectively distinguished liver and pancreatic cancers. The integration of these two glycopeptides, along with the nine tumor markers and 1688 EGPs using a machine learning model enhanced diagnostic accuracy, achieving a receiver operating characteristic‐area under curve (ROC‐AUC) score of 0.996. CSGSA has the potential to minimize the need for invasive diagnostic procedures and serves as a promising tool for widespread screening.

## Graphical abstract

This study presents a novel AI‐based diagnostic approach—comprehensive serum glycopeptide spectra analysis (CSGSA)—that integrates tumor markers and enriched glycopeptides from serum. Using a neural network model, this method accurately distinguishes liver and pancreatic cancers from healthy individuals.

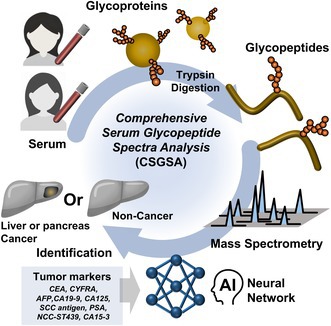

**Abbreviations**
AFP: alpha‐fetoprotein
CA125: cancer antigen 125
CEA: carcinoembryonic antigen
CSGSA: comprehensive serum glycopeptide spectra analysis
HCC: hepatocellular carcinoma
LC–MS: liquid chromatography‐mass spectrometer
PAC: pancreatic cancer
PPV: positive predictive value
PSA: prostate‐specific antigen
QC: quality control

## 1 Introduction

Hepatocellular carcinoma (HCC) and pancreatic cancer (PAC) are responsible for hundreds of thousands of new diagnoses worldwide each year, affecting various racial groups [1]. Despite progress in medical technology, these cancers often result in high mortality rates when detected at advanced stages, primarily due to vague early symptoms and the limitations of conventional diagnostic techniques. In Japan, there are about 37 000 new cases of HCC annually, leading to approximately 25 000 deaths [2]. Similarly, PAC has an annual incidence of around 44 000 cases, resulting in an estimated 38 000 deaths [2]. The 5‐year survival rate for HCC and PAC drops to 2–3% when diagnosed at late stages [2, 3]. In contrast, early detection significantly improves survival rates, with early stage HCC showing survival rates of 37–50%, and PAC reaching about 40%. Although ultrasound is a noninvasive and valuable screening tool for these cancers, it struggles to detect early stage tumors and often cannot differentiate between benign and malignant lesions. Furthermore, the diagnostic accuracy of ultrasound heavily relies on the operator's skill, which limits its availability in areas without sufficiently trained professionals. Blood‐based testing provides a noninvasive and cost‐effective diagnostic option and has the potential to serve as an alternative to ultrasound screening. Alpha‐fetoprotein (AFP) is commonly used for HCC diagnosis [4], but it has limited sensitivity, particularly for early stage detection. PIVKA‐II, which is produced in conditions of vitamin K deficiency or inhibition, enhances early detection capabilities for HCC by complementing AFP's low sensitivity [5, 6]. For PAC, CA19‐9 is the most frequently used serum marker; however, its sensitivity and specificity are limited, particularly in identifying early stage cancer [7], highlighting the need for additional biomarkers to enhance diagnostic accuracy. Despite considerable efforts to identify new blood‐based biomarkers for early cancer detection [8, 9, 10], the effectiveness of individual biomarkers has been constrained. To enhance diagnostic accuracy, combinations of multiple biomarkers have been employed [11, 12, 13]. Additionally, significant advancements have been made by applying machine learning techniques, such as deep learning and ensemble methods, to biomarker analysis [14, 15]. The development of comprehensive analytical methods, including proteomics and metabolomics, has also contributed to this progress [16, 17, 18]. These methodologies have enabled the integration of large biomarker datasets, enhanced the predictive accuracy of diagnostic models, and advanced strategies for early cancer detection.

It is well‐known that aberrant glycan structures on serum proteins emerge during cancer progression [19, 20, 21, 22]. AFP‐L3, a glycosylated isoform of AFP, offers greater specificity for HCC compared to total AFP levels. While AFP levels can be elevated in both benign liver conditions and HCC, AFP‐L3 is more specific to malignant liver tumors [23]. Furthermore, cancer‐related glycosylation changes in haptoglobin have been extensively studied in several cancers, including liver [24] and pancreatic [25] cancers. However, analyzing these glycan modifications remains challenging due to several technical and methodological issues: (i) difficulty in obtaining specific antibodies against aberrant glycans; (ii) the absence of intrinsic fluorescence or ultraviolet absorption in glycans; (iii) low ionization efficiency, which complicates mass spectrometry analysis; and (iv) the need to enzymatically release glycans from their host proteins for thorough analysis.

To address these challenges, we have implemented a proteomic approach that emphasizes the analysis of glycopeptides instead of focusing solely on glycans. This method enables the identification of glycan modifications and alterations in serum protein expression related to cancer progression [26, 27, 28]. However, conventional glycoproteomics encounters significant challenges in effectively separating glycopeptides from nonglycosylated peptides [29]. Although lectins are commonly used for glycopeptide enrichment, no single lectin can selectively bind to all glycan structures, and processing a large number of samples with lectins is still labor‐intensive.

To ensure the practical application of this test, we aimed to develop a simple, robust, and high‐throughput method by tackling two main challenges. First, we enriched glycopeptides based on their differences in molecular weight. When digested with trypsin, glycopeptides are considerably larger than nonglycosylated peptides and can be concentrated using ultrafiltration membranes [28]. Although some nonglycosylated peptides may still contaminate the sample, this approach significantly increased throughput compared to lectin‐based enrichment. Second, instead of trying to identify all glycopeptides detected by mass spectrometry, we focused on key glycopeptides that were essential for the discriminative model. We chose this approach because determining the structure of glycopeptides is much more complex than determining peptides in proteomics. These optimizations greatly enhanced our analytical efficiency, enabling us to process over 1000 samples [30].

In this study, we aim to achieve two primary objectives: first, to identify novel glycan biomarkers linked to the onset of liver and pancreatic cancers from over 10 000 enriched glycopeptides (EGPs) [26, 27, 28, 31], and second, to develop a robust cancer detection model that combines EGPs and conventional tumor markers using advanced machine learning techniques. This method, known as *comprehensive serum glycopeptide spectra analysis* (CSGSA), integrates more than 1000 EGPs with conventional tumor markers through machine learning [30, 32, 33, 34]. CSGSA is anticipated to significantly lower both false‐positive and false‐negative rates by merging clinically validated tumor markers, which have limited sensitivity, with glycan structures that are highly responsive to cancer development. To evaluate the practical effectiveness of this newly developed model for cancer screening, we assessed its performance using receiver operating characteristic‐area under curve (ROC‐AUC) and prevalence‐adjusted positive predictive value (PPV).

## 2 Materials and methods

### 2.1 Study design

This study was structured as a retrospective, observational, case–control study. All patients and healthy volunteers who visited the hospital during a designated period were considered for inclusion. Random sampling and blinding were not employed. Based on an assumed alpha error of 0.05 and a beta error of 0.2, and anticipating that the average expression levels of the target markers would differ by about half of the standard deviation between the cancer and healthy groups, the minimum required sample size was estimated to be approximately 100. We enrolled 119 patients with PAC, 49 with HCC, and 590 healthy volunteers (Table 1). Serum samples were collected at the time of cancer diagnosis, before any treatment or surgical intervention. The sera from Japanese patients were sourced from our hospital, the National Hospital Organization Kyushu Medical Center (Fukuoka, Japan).

**Table 1:** Demographic characteristics of the patients. The numbers in the parentheses indicate the standard deviation of the age or number of participants.

Condition	Age	Number	Sex (man ratio)	Stage	Race
Healthy Volunteers (HE)	48.2 (±12.2)	590	50.3%		Asian (297) Caucasian (114) African American (115) Hispanic (63) Mixed Ethnicities (1)
Pancreatic Cancer (PAC)	66.1 (±8.6)	119	43.6%	Stage I (16) Stage II (44) Stage III (17) Stage IV (35) Unclassified (7)	Asian (41) Caucasian (78)
Hepatocellular Carcinoma (HCC)	71.0 (±9.2)	49	67.3%	Stage I (2) Stage II (29) Stage III (12) Stage IV (2) Unclassified (4)	Asian (49)
Total	52.2 (±14.1)	758	50.4%		

Serum samples from Caucasian cancer patients were sourced from KAC Corporation (Kyoto, Japan) and Sunfco Ltd. (Tokyo, Japan). The sera of healthy Japanese volunteers were obtained from LSI Medience Corporation (Tokyo, Japan) and SOIKEN (Osaka, Japan), while samples from Caucasian, African American, and Hispanic individuals were collected from KAC Corporation and Sunfco Ltd. Written informed consent was obtained from all patients and volunteers. The Institutional Review Boards of the National Hospital Organization Kyushu Medical Center (IRB registration number: 17C299; December 20, 2017) and LSI Medience Corporation (IRB registration number: MS/Shimura 17–19; January 22, 2018) approved the use of patient clinical information and serum samples.

All research methods and procedures strictly followed the ethical principles outlined in the Declaration of Helsinki and fully complied with applicable institutional, national, and international guidelines. The inclusion criteria for this study were as follows: (i) patients diagnosed with primary cancer through imaging or histological analysis; (ii) patients at the time of initial diagnosis or cancer recurrence who had not yet started treatment; and (iii) patients aged 20 years or older at the time of consent. The exclusion criteria included (i) patients with severe renal, hepatic, respiratory, or cardiac dysfunction, or those with concurrent infectious diseases; (ii) patients considered unsuitable for study participation by the attending physician; and (iii) patients who had already begun any form of treatment.

Cancer staging was conducted using the TNM classification system set by the Union for International Cancer Control (UICC) [35]. Blood samples were collected through venous puncture before surgery or treatment. Sera were separated from blood cells by centrifugation within 8 h of collection and stored at −80 °C until analysis. Each sample was analyzed only once, as their accuracy and reproducibility had been previously validated.

### 2.2 Tumor marker analyses

Nine tumor markers—carcinoembryonic antigen (CEA), carbohydrate antigen 19–9 (CA19‐9), cytokeratin 19 fragment (CYFRA), NCC‐ST439, cancer antigen 125 (CA125), prostate‐specific antigen (PSA), cancer antigen 15–3 (CA15‐3), alpha‐fetoprotein (AFP), and squamous cell carcinoma antigen (SCC antigen)—were analyzed by LSI Medience Corporation, a clinical testing laboratory based in Tokyo, Japan.

### 2.3 Sample preparation and liquid chromatography–tandem mass spectrometry

The sample preparation and analysis procedures were adapted from our previous work [34], with the following modifications: A 20 μL aliquot of serum was mixed with 120 μL of acetone containing 10% trichloroacetic acid to precipitate proteins. The precipitate was then mixed with a denaturing solution, made of urea (80 μg, Wako Pure Chemical Industries), Tris/HCl buffer (pH 8.5, 100 μL), 0.1 M EDTA solution (10 μL), 1 m Tris (2‐carboxyethyl) phosphine hydrochloride (5 μL, Sigma), and water (38 μL) and incubated for 10 min at 37 °C to denature the proteins. Next, 1 m 2‐iodoacetamide (40 μL, Wako Pure Chemical Industries) was added to the denaturing solution to protect the thiol residues in the proteins, and the mixture was kept in the dark for 10 min at 37 °C. The mixture was then transferred to a 30 kDa ultrafiltration tube (Amicon Ultra 0.5 mL, Millipore Corp., MA, USA) to remove the denaturing agents. Protein digestion was performed on the filter using 200 μL of 0.1 m Tris/HCl buffer (pH 8.5), 20 μL of 0.1 μg·μL^−1^ trypsin (Wako Pure Chemical Industries, Osaka, Japan), and 20 μL of 0.1 μg·μL^−1^ lysyl endopeptidase (Fujifilm Wako Pure Chemical Industries), followed by incubation for 16 h at 37 °C. After digestion, the mixture was centrifuged at 11 500 ***g*** for 30 min. The resulting filtrate, which contained both digested peptides and glycopeptides, was then transferred to a 10 kDa ultrafiltration tube (Amicon Ultra 0.5 mL, Millipore Corp.) to separate glycopeptides from nonglycosylated peptides [28]. The compounds retained by the 10 kDa ultrafiltration, referred to as enriched glycopeptides (EGPs), were subsequently analyzed using liquid chromatography coupled with a quadrupole time‐of‐flight mass spectrometer (HP1200 + 6520, Agilent Technologies, Palo Alto, CA, USA), equipped with a C18 column (Inertsil ODS‐4, 2 μm, 100 Å, 100 mm × 2.1 mm ID, GL Science, Tokyo, Japan). The EGPs were eluted using a gradient program at a flow rate of 0.2 mL·min^−1^ and a column temperature of 40 °C: starting with 15% to 30% mobile phase B over 7 min, increasing to 30% to 50% from 7 to 12 min, followed by a 2‐min hold at 100% mobile phase B. Mobile phase A contained 0.1% formic acid in water, while mobile phase B consisted of 0.1% formic acid in 9.9% water and 90% acetonitrile. The mass spectrometer was operated in negative ion mode with a capillary voltage of 4000 V. Each sample was analyzed once. Negative ion mode was employed to enhance glycopeptide signal intensity and suppress nonglycosylated peptide interference. Due to the presence of sialic acids, many glycopeptides ionize more efficiently in negative mode, whereas coexisting nonglycosylated peptides tend to dominate in positive mode. This polarity selection improved glycopeptide signal‐to‐noise ratios for scan‐based profiling. In total, 1688 EGPs were selected from over 10,000 detected peaks through a three‐step process: (i) removing peaks with low reproducibility (coefficient of variation (CV) >50%), (ii) eliminating peaks with low reliability (S/N < 5), and (iii) excluding isotope, adduct, and fragment ions. The remaining 1688 EGP peaks were then used for biomarker screening and CSGSA diagnostics.

### 2.4 Data processing

The data processing methods are described in previous publications [26, 28]. In summary, the raw data from liquid chromatography‐mass spectrometry (LC–MS) were exported in CSV format using Mass Hunter Export software (Agilent Technologies). Peak positions (retention times and m/z values) and peak areas were extracted using R software (R 3.2.2, R Foundation). Marker Analysis software (LSI Medience Corporation, Tokyo, Japan) was utilized to align peak areas, reduce noise, and correct discrepancies [28]. The tolerances for peak alignment and assignment for m/z and retention time were set at 0.06 Da and 0.3 min, respectively. The relative expression of 1688 EGPs for each sample was determined by calculating expression ratios against to a quality control standard. The dataset was randomly divided into two subsets: 70% for training and 30% for validating. A model was trained using the training set, and its accuracy was evaluated with the validation set. This procedure was repeated 10 times to ensure the model's robustness. The results from each iteration were combined, and the ROC‐AUC was calculated from the cumulative results. Model outputs, which ranged from 0 to 1, were then converted into CSGSA scores using the following formula:

$$\text{CSGSA score}=-\log_{10}\;\left(1-\text{predicted value}\right)$$

This transformation provided a score that is easier to interpret for clinical and diagnostic purposes.

To rigorously evaluate the diagnostic performance of Model 3, we implemented the following procedure. First, we predefined a hold‐out test set by excluding the following samples from the full cohort: 50 healthy controls collected by SOIKEN between 2011 and 2018, 14 patients with PAC collected by Sunfco between 2011 and 2018, and 8 patients with HCC collected by KMC between 2011 and 2018. The remaining samples were used to develop the model. Specifically, 70% of the nonhold‐out samples were used as the training set, and the remaining 30% were used as the internal validation set. The model was trained on the training set and evaluated on the internal validation set, and this process was repeated 20 times with different random splits. After aggregating the results from the 20 internal validation sets to assess overall performance, the finalized model was applied to the hold‐out test set for independent evaluation (Tables S1 and S2, Fig. S1).

### 2.5 Identification of the glycopeptide features contributing to cancer discrimination

To identify putative glycopeptide features, we compared the retention times, accurate mass spectra, and isotopic distribution patterns of glycopeptides from patient serum with those derived from commercially available purified human serum proteins. The reference proteins included alpha‐1‐acid glycoprotein, complement C8, complement C9, complement factor H, fibrinogen, haptoglobin, alpha‐2‐macroglobulin, antitrypsin, and transferrin, all obtained from Sigma‐Aldrich (St. Louis, MO, USA). For each reference protein, the amino acid sequence was obtained from the UniProt database, and theoretical tryptic peptides (no missed cleavages) were generated. Among these, only peptides containing the N‐X‐S/T consensus motif—known sites for N‐linked glycosylation—were considered. Known glycosylation sites annotated in UniProt were also referenced. These peptides were then computationally combined with a library of plausible N‐glycan compositions typically found in human serum, including bi‐, tri‐, tetra‐, and penta‐antennary glycans with varying levels of fucosylation (0–6 residues) and the presence or absence of sialic acids. The resulting theoretical glycopeptides were used to match features detected in patient samples based on m/z values (within a ± 0.03 Da tolerance), retention times, and isotopic patterns. Since glycopeptides with the same peptide backbone but different glycans tend to co‐elute in reversed‐phase LC, retention time served as a useful secondary filter. When a match was established with one glycoform, related glycopeptide features with the same peptide and different glycan compositions could be inferred. The high‐resolution TOF‐MS data combined with retention time and isotopic pattern matching enabled us to tentatively assign glycopeptide features with high confidence.

### 2.6 Statistical analysis

The machine learning model was developed using Python (version 3.12, 64‐bit). To compare EGP levels between cancer patients and healthy controls, we used the Student's *t*‐test, assuming a parametric distribution for all EGPs. Missing data, mainly due to values falling below the detection threshold, were replaced with 0 s.

Model performance was evaluated using a 70/30 train/validation split, repeated independently 10 times. In each iteration, training and validation sets were mutually exclusive, and no sample was used for both training and validating within the same run. This approach allowed us to assess the stability and robustness of the model across multiple random partitions.

Comprehensive statistical analyses were conducted using SPSS (version 27.0, Chicago, IL, USA) along with proprietary software [28]. Principal component analysis (PCA) was performed using SIMCA software (version 13.0.3; Umetrics). ROC curve analyses and AUC calculations were performed using the roc_auc_score and roc_curve functions from the scikit‐learn package in Python, based on the predicted probabilities generated by the neural network model. The true positive rate (TPR, sensitivity) and false positive rate (FPR, 1‐specificity) were computed by comparing the predicted class probabilities against the known true labels across various decision thresholds.

## 3 Results

### 3.1 Comparison of levels of nine tumor markers in patients with PAC, HCC, and healthy volunteers

**Fig. 1:**
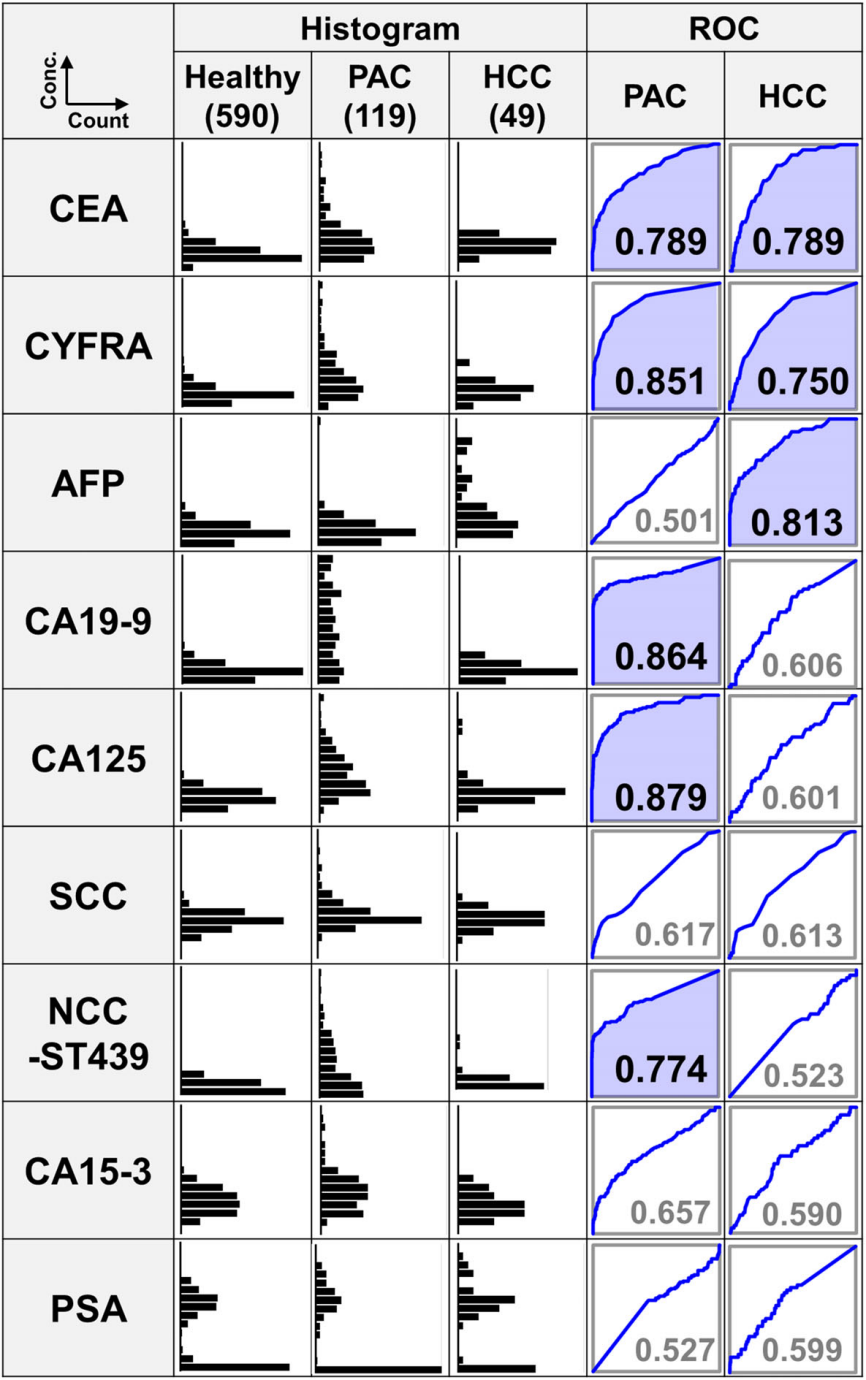
Levels of tumor markers in PAC and HCC patients, and healthy volunteers. This figure shows the levels of nine tumor markers: CEA, CYFRA, AFP, CA19‐9, CA125, SCC antigen, NCC‐ST439, CA15‐3, and PSA, in individuals with PAC, HCC, and healthy volunteers. Histograms represent the distribution of logarithmically transformed marker levels (log base 10) on the vertical axis against the number of individuals on the horizontal axis. ROC curves are included to compare the diagnostic performance of each marker between the cancer groups (PAC or HCC) and the healthy group, with AUC values indicated. Curves with AUC values above 0.7 are highlighted in blue.

The levels of nine tumor markers in patients with PAC, HCC, and healthy volunteers are shown in histograms (Fig. 1). All values were transformed using a logarithmic scale, and receiver operating characteristic (ROC) analysis was performed for each cancer group compared to the healthy group. ROC curves with an area under the curve (AUC) greater than 0.7 are highlighted in blue. Notably, carcinoembryonic antigen (CEA) levels were significantly elevated in patients with both PAC and HCC (AUC = 0.789 for both). Interestingly, CYFRA, which is typically used for diagnosing nonsmall cell lung cancer, also showed significant increases in PAC and HCC (AUC = 0.851 and 0.750, respectively). CYFRA is a fragment of cytokeratin 19 found in epithelial cells that degrades during cell death and enters the bloodstream [36]. This suggests that CYFRA may have broader diagnostic applications beyond lung cancer, potentially serving as a valuable biomarker for PAC and HCC detection. As expected, AFP, a marker for HCC, and CA19‐9 for PAC, showed increases in patients with their respective cancers (AUC = 0.813 and 0.864, respectively). Other tumor markers, such as SCC antigen (linked to lung and uterine cancers), PSA (prostate cancer), and CA15‐3 (breast cancer), did not show elevated levels in PAC or HCC, indicating high specificity. Notably, a significant increase in CA125 levels, which is typically used for diagnosing ovarian cancer, was found in PAC cases (AUC = 0.879). This observation may offer valuable insights for the identification of PAC.

### 3.2 Overview of EGP alterations in PAC and HCC

**Fig. 2:**
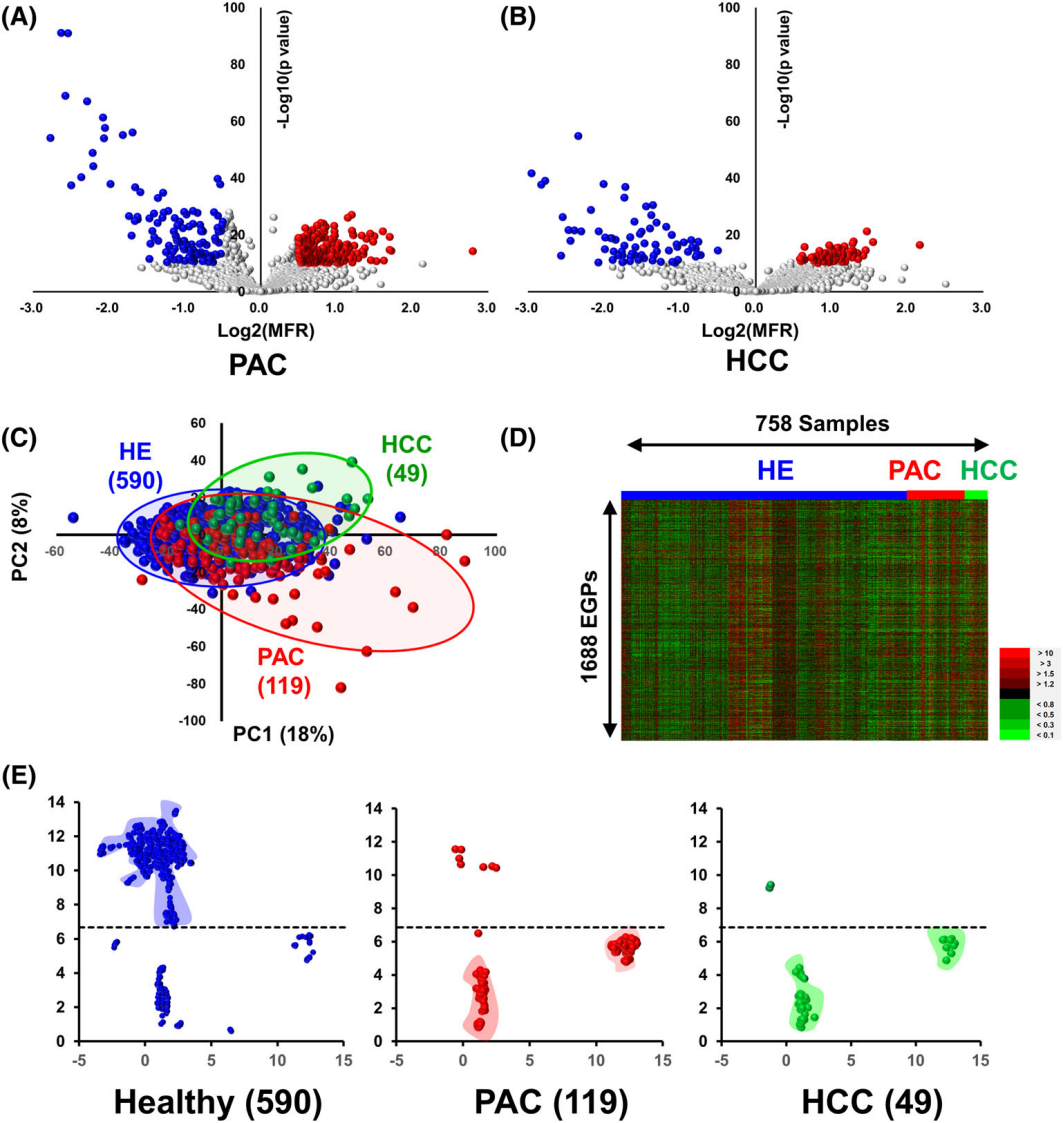
Overview of EGP expressions in PAC and HCC. (A) Volcano plot comparing PAC to healthy groups. The vertical axis shows the negative logarithm (base 10) of the *P*‐value from the Student's *t*‐test, and while the horizontal axis displays the logarithm (base 2) of the mean fold ratio (MFR). EGPs with a *P*‐value less than 10^−10^ and an MFR greater than 2^0.5^ are marked in red, and those with a *P*‐value less than 10^−10^ and an MFR less than 2^−0.5^ are marked in blue. (B) Volcano plot comparing HCC to healthy groups. (C) Score plot from PCA representing data for PAC (red), HCC (green), and healthy (blue) groups. (D) Heatmap illustrating the expression profiles of 1688 EGPs across 758 individuals, where overexpressed EGPs are shown in red and downregulated EGPs are shown in green. (E) Analysis of UMAP for healthy (blue), PAC (red) and HCC (green) groups.

We extracted 1688 EGPs in the sera of patients with PAC and HCC, which serve as robust and reliable markers. Volcano plots indicate that EGP levels in the sera of patients with PAC changed dramatically, with many of them significantly reduced compared to those in HCC (Fig. 2A,B). PCA showed that the data distributions for PAC and HCC slightly deviated from healthy controls, highlighting more significant glycan changes in these cancer groups (Fig. 2C). While the heatmap did not clearly differentiate between the cancer groups and healthy controls (Fig. 2D), uniform manifold approximation and projection (UMAP), a technique for visualizing high‐dimensional data [37], displayed distinct distribution patterns among the healthy, PAC, and HCC groups. Most healthy individuals clustered at the upper side, while patients with PAC and HCC were primarily located at the lower side (Fig. 2E).

### 3.3 Identification of novel cancer‐specific biomarkers in EGPs

**Fig. 3:**
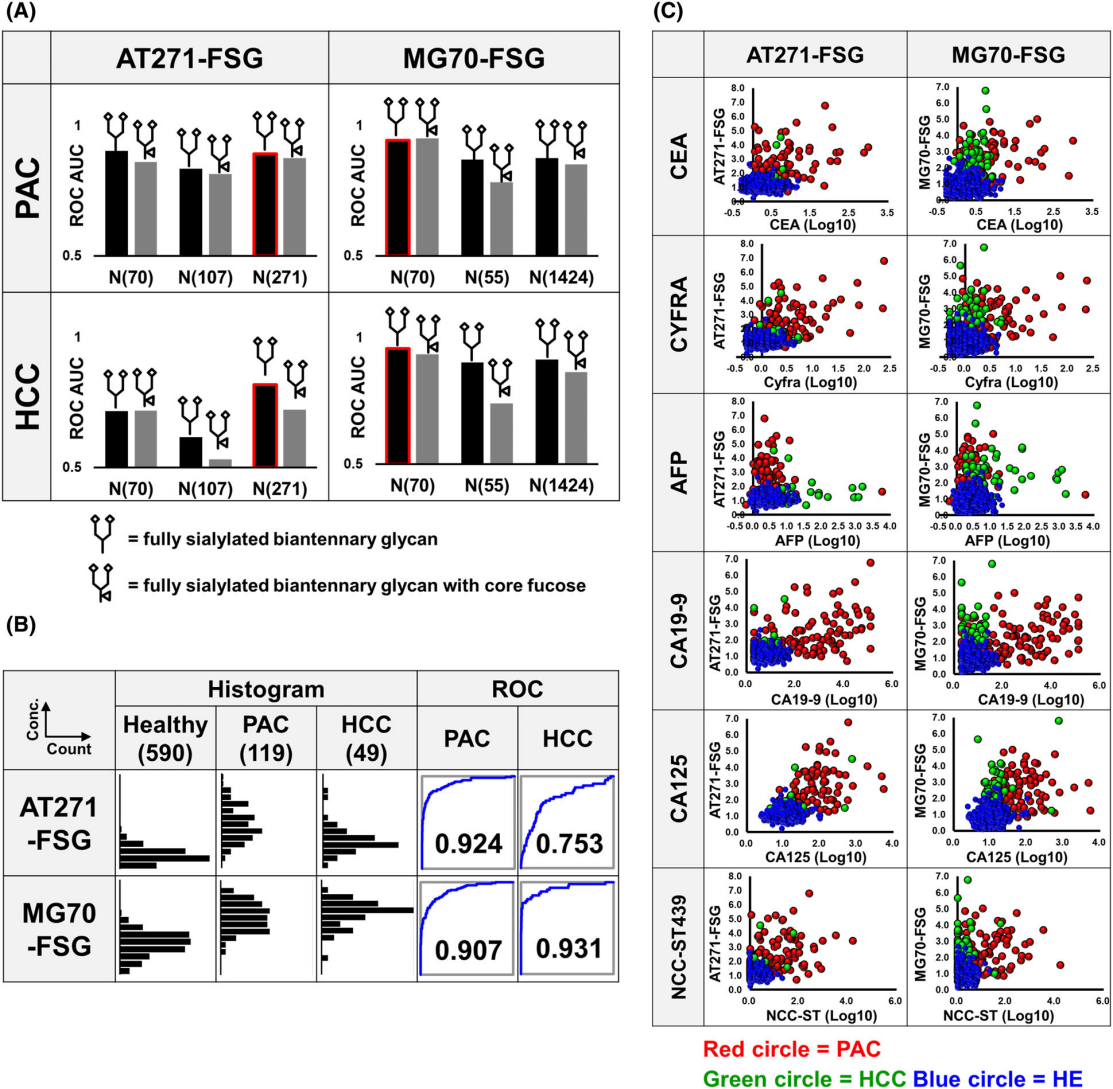
Analysis of α1‐antitrypsin and α2‐macroglobulin‐derived glycopeptides. (A) ROC‐AUC values for glycopeptides derived from α1‐antitrypsin (AT) and α2‐macroglobulin (MG), with glycopeptides used in machine learning outlined in red. The numbers following ‘N’ denote the position of asparagine sequence from the N‐terminal. (B) Histograms illustrate the distribution of AT271‐FSG and MG70‐FSG levels across PAC, HCC, and healthy groups. Accompanying ROC curves demonstrate the diagnostic accuracy of each glycopeptide, with AUC values provided for distinguishing between cancer and healthy groups. (C) Scatter plots depict the correlation between glycopeptide levels (AT271‐FSG and MG70‐FSG) and tumor markers (CEA, CYFRA, AFP, CA19‐9, CA125, and NCC‐ST439) associated with PAC or HCC. The vertical axes represent the levels of AT271‐FSG or MG70‐FSG, while the horizontal axes display the logarithmically transformed levels of the tumor markers.

Through our extensive analysis of nearly 10 000 EGPs, we identified several promising biomarker candidates that showed statistically significant differences between cancer patients and healthy controls. Candidates were selected based on very low *P*‐values (below 10^−10^) from Student's t‐tests and a mean fold change greater than 1.5. To ensure precise quantification, EGP expression levels were normalized against transferrin levels, which serve as a reliable endogenous internal standard due to their stable expression across samples, and minimize variability from sample collection and preparation. To develop a robust screening test, glycopeptides with low measurement reproducibility among the extracted candidates were excluded. This process led to the identification of two glycopeptides, α1‐antitrypsin (AT) and α2‐macroglobulin (MG), which effectively distinguish PAC and HCC from healthy groups. Analyzing their glycan modifications and attachment sites revealed specific impacts on the ROC‐AUC; notably, glycan chains attached to asparagine at position 271 on α1‐antitrypsin and position 70 on α2‐macroglobulin significantly impacted diagnostic discrimination (Fig. 3A). Although several glycans were detected on both AT and MG, only the fully sialylated biantennary glycan and the one with core fucose were evaluated, as these provided reliable quantitative measurements. While the differences between the two glycopeptides were minimal, the ROC‐AUC for the fully sialylated biantennary glycan slightly surpassed that of the one with core fucose. The ROC‐AUC for α1‐antitrypsin with a fully sialylated biantennary glycan linked to asparagine 271 (AT271‐FSG) reached 0.924 in PAC, but its response in HCC was lower (0.753). In contrast, the ROC‐AUC for α2‐macroglobulin with a fully sialylated biantennary glycan linked to asparagine 70 (MG70‐FSG) achieved 0.907 in PAC and 0.931 in HCC, demonstrating strong performance similar to that of conventional tumor markers (Fig. 3B). Figure 3C illustrates the relationships between AT271‐FSG and MG70‐FSG and other tumor markers, including CEA, CYFRA, AFP, CA19‐9, CA125, and NCC‐ST439, which all showed significant responses to PAC or HCC. While slight positive correlations were noted, they were not strong, suggesting that combining these markers could further enhance diagnostic accuracy for PAC and HCC.

### 3.4 Discriminant modeling of PAC and HCC by machine learning using tumor markers, AT271‐FSG, MG70‐FSG, and 1688 EPGs

**Fig. 4:**
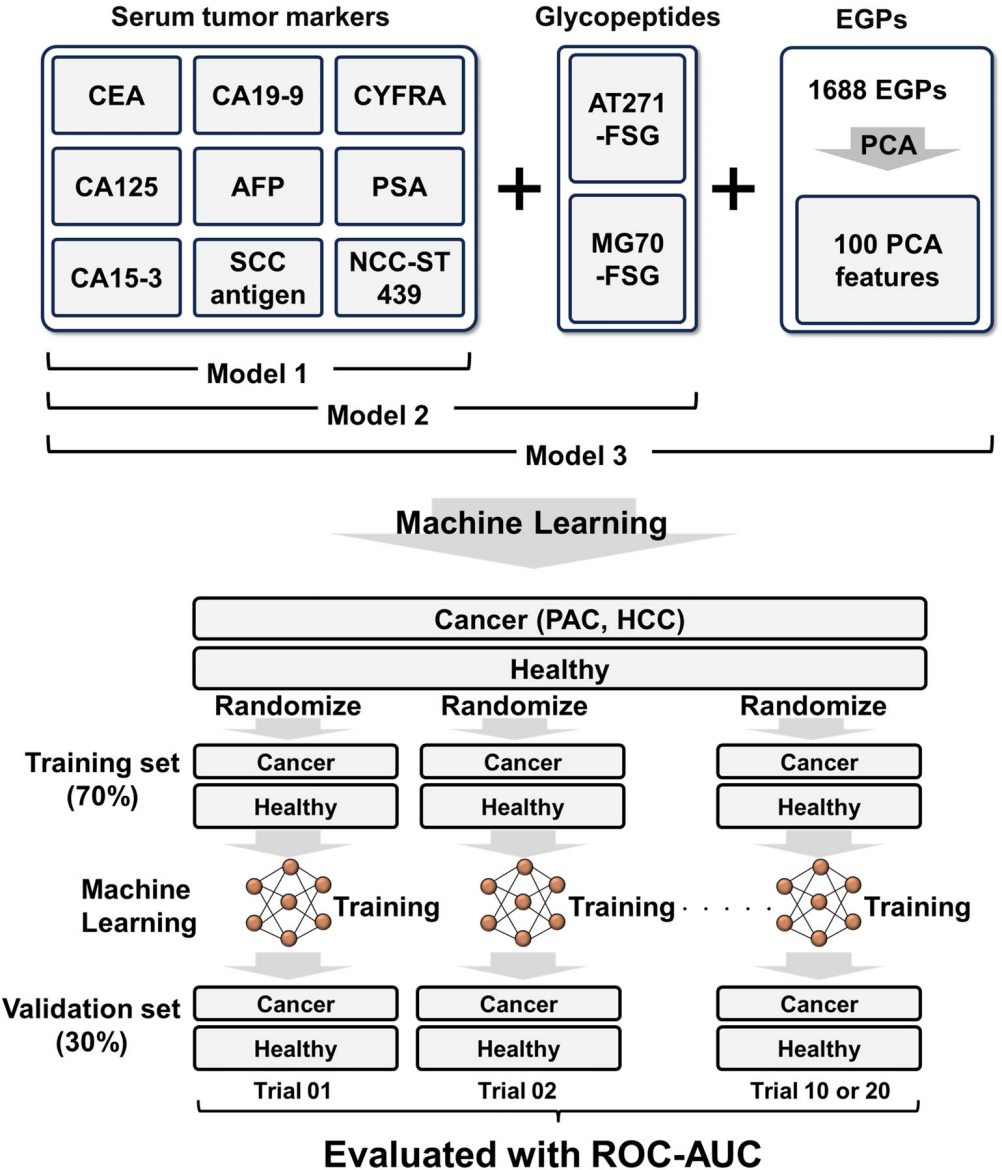
Machine learning model development and evaluation strategy. This figure illustrates the development and evaluation of three distinct machine learning models to assess how different biomarker sets affect the accuracy of cancer diagnosis. Model 1 uses nine conventional serum tumor markers, Model 2 adds glycopeptides linked to α1‐antitrypsin (AT271‐FSG) and α2‐macroglobulin (MG70‐FSG), and Model 3 combines these elements with 100 key glycan features obtained from PCA of 1688 EGPs. Each model was trained on a randomly selected training set comprising 70% of the samples, while the remaining 30% was designed as the validation set. The evaluation was repeated 10 times to ensure consistency, and the results were collectively analyzed using ROC analysis to assess performance across different configurations.

**Fig. 5:**
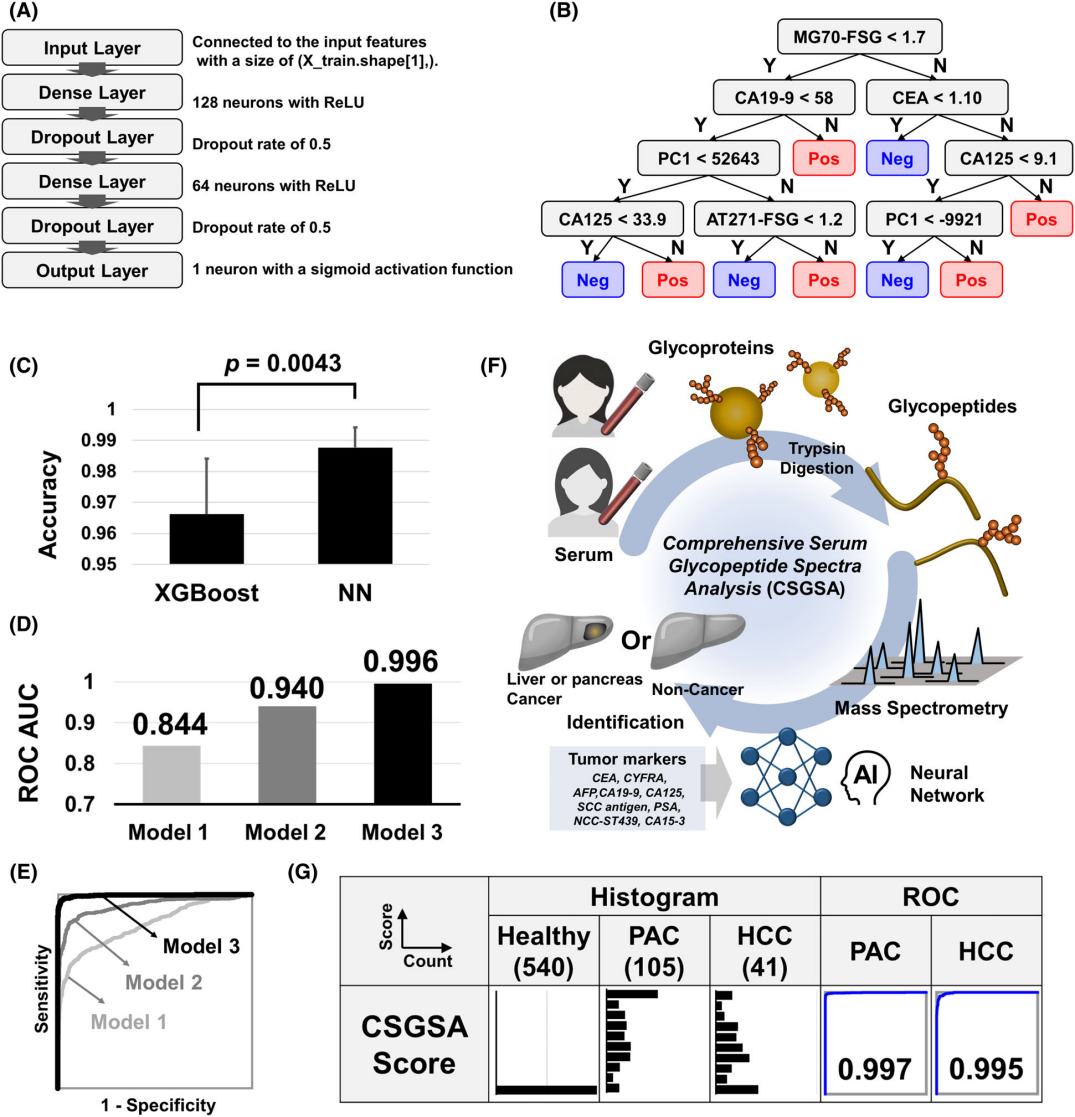
Comprehensive evaluation of machine learning models and CSGSA Score. (A) Structure of the neural network model: The neural network includes an input layer, followed by two sets of dense and dropout layers, and concludes with an output layer. The dropout layers help prevent overfitting by randomly setting a portion of input units to zero during training. (B) Example of a tree structure optimized by XGBoost: The tree structure, particularly in the lower nodes, varies with each training set selection. (C) Performance comparison of cancer identification using Model 3 with XGBoost and neural network algorithms, highlighting accuracy. The *P*‐value was calculated using the Student's *t*‐test. The error bars represent standard deviations. (D) ROC‐AUC values for each model, illustrating their effectiveness in cancer identification. (E) The ROC curves for three different models. (F) CSGSA workflow: This illustration shows the detection model for PAC and HCC cancers, combining 9 tumor markers, AT271‐FSG, MG70‐FSG, and 1688 EGPs. (G) CSGSA score distribution and performance: Histograms display the distribution of CSGSA scores across different groups (PAC, HCC, and healthy). ROC curves evaluate the diagnostic performance of Model 3.

To enhance diagnostic accuracy, we developed a comprehensive machine learning model that combines conventional tumor markers with AT271‐FSG, MG70‐FSG, and 1688 EGPs. We assessed three models to clarify the specific contributions of these biomarkers: Model 1 utilized only nine tumor markers; Model 2 added AT271‐FSG and MG70‐FSG to nine tumor markers; and Model 3 incorporated all markers along with 100 key features derived from the 1688 EGPs using PCA. Although both AT271‐FSG and MG70‐FSG are glycopeptides, they were treated as independent variables to highlight their unique diagnostic potential. The reduction of features from 1688 to 100 through PCA aimed to prevent overfitting, a common issue in machine learning. We trained the model on 70% of randomly selected samples and evaluated its performance on the remaining 30%. This process was repeated 10 times to ensure robustness, and the aggregated results were analyzed using ROC analysis (Fig. 4). In the development of Model 3, we developed both XGBoost and neural network architectures. XGBoost, an advanced implementation of gradient boosting algorithms, excels in classification and regression tasks by creating an ensemble of weak prediction models, typically decision trees [38]. In contrast, neural networks employ layers of interconnected nodes to model complex patterns in data and classify samples [39]. Figure 5A shows the neural network architecture, consisting of an input layer, two pairs of dense and dropout layers, and an output layer, with the dense layers limited to two to prevent overfitting. Figure 5B depicts the typical tree structure optimized by XGBoost, highlighting MG70‐FSG, CA19‐9, CEA, CA125, and AT271‐FSG as key factors in differentiating PAC and HCC. The tree structure varied based on the training set used, particularly influencing the lower layers' configuration. A comparison of the performance of the two models revealed that the neural network model outperformed XGBoost in differentiating cancer groups from healthy controls (Fig. 5C). The ROC‐AUC scores for differentiating cancer groups from healthy group were 0.844 (Model 1), 0.940 (Model 2), and 0.996 (Model 3), significantly exceeding those of current tumor markers (Fig. 5D,E).

The workflow of the CSGSA, utilizing machine learning Model 3, is shown in Fig. 5F. In the CSGSA screening test, the predicted values from Model 3 were converted into CSGSA scores (ranging from 0 to 10) using the following formula:

$$\text{CSGSA score}=-\log_{10}\left(1–\text{Model}\;3\;\text{predicted value}\right).$$

Figure 5G displays histograms based on the CSGSA scores for PAC and HCC, demonstrating that CSGSA effectively differentiated PAC and HCC from the healthy group, with ROC‐AUC values of 0.997 and 0.995, respectively. A cutoff value of 1 was used to classify samples into positive and negative groups, resulting in sensitivities of 93.6% for PAC and 79.2% for HCC, while specificities exceeded 99.9% for both cancers (Table 2). When the model was applied to the hold‐out test set, all 50 healthy controls and all 14 patients with PAC were correctly classified as negative and positive, respectively. In contrast, among the 8 patients with HCC, 62.5% (5/8) were correctly identified as positive (Table 3). This cutoff was strategically set to reduce false positives rather than false negatives, aiming to improve the PPV, which is essential for an effective screening test. The PPVs for PAC and HCC were 34.7% and 27.9%, respectively. The lower‐than‐expected PPV is due to the low prevalence of these cancers, with PAC occurring in 35 cases per 100 000 individuals and HCC in 30 cases per 100 000 individuals [40].

**Table 2:** Evaluation of the cancer screening model using validation set.

(A) Patients with pancreatic cancer and healthy individuals						
			True state			
			PAC	Healthy	Sum	PPV or NPV
Predicted state	Observed samples	Positive	586	2	588	
		Negative	40	3233	3273	
		Sum	626	3235	3861	
	Prevalence correction	Positive	33	62	95	34.7%
		Negative	2	99 903	99 905	99.998%
		Sum	35	99 965	100 000	
	Sensitivity or specificity		93.6%	99.94%		

(B) Patients with hepatocellular carcinoma and healthy participants						
			True state			
			HCC	Healthy	Sum	PPV or NPV
Predicted state	Observed samples	Positive	205	2	207	
		Negative	54	3233	3287	
		Sum	259	3235	3494	
	Prevalence correction	Positive	24	62	86	27.9%
		Negative	6	99 908	99 914	99.994%
		Sum	30	99 970	100 000	
	Sensitivity or specificity		79.2%	99.94%		

**Table 3:** Evaluation of the cancer screening model using hold‐out test set.

(A) Patients with pancreatic cancer and healthy individuals						
			True state			
			PAC	Healthy	Sum	PPV or NPV
Predicted state	Observed samples	Positive	14	0	14	
		Negative	0	50	50	
		Sum	14	50	64	
	Prevalence correction	Positive	35	0	95	100.0%
		Negative	0	99 965	99 965	100.000%
		Sum	35	99 965	100 000	
	Sensitivity or specificity		100.0%	100.00%		

(B) Patients with hepatocellular carcinoma and healthy participants						
			True state			
			HCC	Healthy	Sum	PPV or NPV
Predicted state	Observed samples	Positive	5	0	5	
		Negative	3	50	53	
		Sum	8	50	58	
	Prevalence correction	Positive	19	0	19	100.0%
		Negative	11	99 970	99 981	99.989%
		Sum	30	99 970	100 000	
	Sensitivity or specificity		62.5%	100.00%		

### 3.5 Enhancing cancer type classification through neural network modeling

**Fig. 6:**
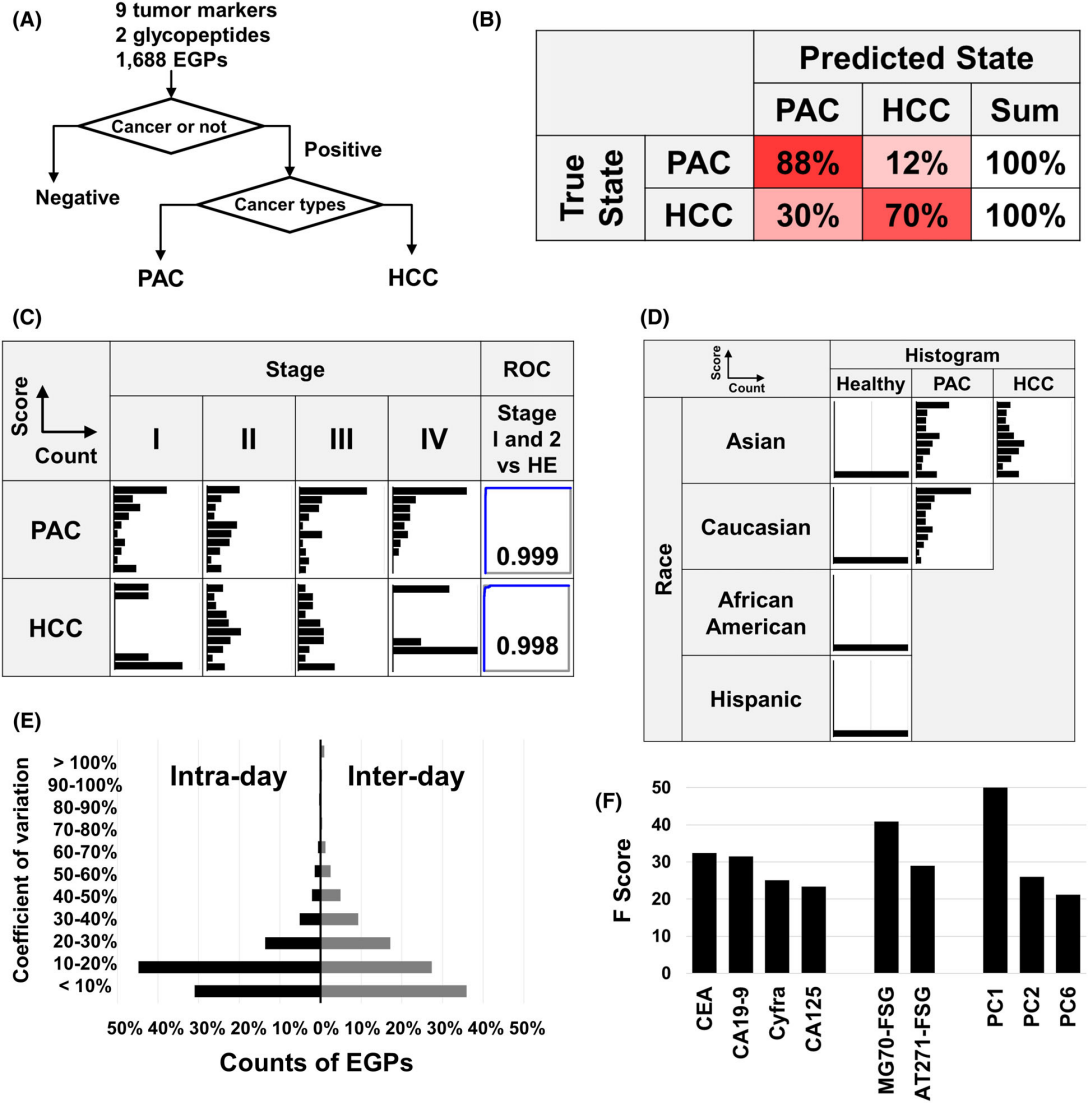
Evaluation of CSGSA. (A) Classification strategy: This outlines the analytical process in which individuals are first classified as cancerous or noncancerous, followed by further classification into specific cancer types (PAC and HCC). (B) Prediction accuracy: This section summarizes the accuracy of predictions for each cancer type, showing the percentage of correct identifications relative to the total predictions made. (C) Staging analysis: Histograms illustrate the distribution of CSGSA scores across different cancer stages. ROC analysis compares stage I and II to the healthy group, indicating the method's ability for early detection. (D) Relationship between CSGSA scores and race. Histograms show CSGSA scores for healthy individuals and patients with PAC and HCC in Asian and Caucasian populations. The African American and Hispanic groups are represented in histograms for healthy individuals only. (E) Intraday and interday reproducibility of 1688 EGPs: Each EGP was measured five times within the same day (considering preparation and MS measurement errors), and the CV for each EGP was calculated and presented as a histogram. For interday reproducibility, measurements were taken five times per day, and the average of these values were obtained. This process was repeated over 3 days with the variation (CV values) of the mean values (*n* = 3) shown in a histogram. (F) Contributors to Model 3 identified through XGBoost's F Scores, visualizing the contribution of each factor to the model.

To enhance the classification accuracy of liver and pancreatic cancers among positively identified patients, we implemented an additional neural network‐based classification model (Fig. 6A). This model was systematically trained using 70% of the patients with cancers as the training set and thoroughly evaluated on the remaining 30% as the validation set. To ensure robustness, this process was repeated 10 times and the combined results were analyzed using ROC analysis. Our results revealed a classification accuracy of 88% for PAC and 70% for HCC (Fig. 6B). The significantly lower performance in HCC classification is mainly due to the small sample size, which was insufficient to fully capture the complexity of the disease's biomarker profile.

### 3.6 Relationship between CSGSA score and cancer development (stage)

We examined the relationship between CSGSA scores and cancer staging. When the CSGSA scores were categorized into four stages—I, II, III, and IV—it was noted that the CSGSA scores increased progressively with advancing stages in PAC, while no significant changes were observed in HCC (Fig. 6C). We compared patients in stages I and II, classified as early stage, with a healthy control group. The ROC‐AUC values were 0.999 for PAC and 0.998 for HCC, highlighting the potential of the CSGSA test in detecting early stage cancers.

### 3.7 Relationship between CSGSA scores and race

We examined the relationship between race and CSGSA scores using histograms (Fig. 6D). The results showed no significant differences between Asians and Caucasians. In healthy individuals, scores were generally below 1, while an increase in scores was observed in patients with PAC. Caucasians exhibited a more pronounced increase compared to Asians, likely because the study included a higher proportion of advanced‐stage cases in the Caucasian group. African Americans and Hispanics were analyzed only among healthy individuals, and their scores were also below 1, similar to those of Asians and Caucasians. These findings suggest that the CSGSA method consistently reflects cancer presence across different ethnicities.

### 3.8 Method validation

To ensure the reproducibility of glycopeptide analysis, the entire process from pretreatment to LC–MS measurement was repeated five times in one day using five aliquots of serum from a cancer patient, and this procedure was repeated over three consecutive days. The coefficient of variation (CV) for intraday reproducibility was calculated from the five measurements taken on the first day, while interday reproducibility was assessed by determining the CV of the daily averages across 3 days. The intraday CVs for AT271‐FSG and MG70‐FSG were 4.8% and 9.1%, respectively, while interday CVs were 3.3% and 7.5%. More than 80% of EGPs had a CV of less than 30% for both intraday and interday reproducibility (Fig. 6E). We have previously reported on (i) the stability of glycoproteins from blood collection to serum separation; (ii) their storage stability postserum separation; (iii) the diurnal variation of glycoproteins, including dietary effects; and (iv) intermachine variability in mass spectrometry. These evaluations confirmed that glycoproteins are stable under certain conditions [30].

### 3.9 Key contributors to the model efficacy

Identifying the key contributors to the neural network model (Model 3) is crucial; however, the neural network framework limits insights into which explanatory variables affect the model's outcomes. In contrast, XGBoost enables the identification of these contributions; therefore, we used it to estimate the contributing factors, albeit indirectly. In XGBoost, the ‘F Score’ effects how often a feature is used to split the data across all trees in the model. A higher F Score indicates that the feature plays a more significant role in a model construction and is crucial to the decision‐making process. Our findings showed that CEA, CA19‐9, and CYFRA were significant contributors (Fig. 6F), supported by their strong ROC‐AUC performances (Fig. 1). Notably, CA125 also significantly impacted on the model, primarily due to its increase in the PAC group. Among the features derived from PAC, PC1 was particularly influential, as evidenced by the slight separation between the cancer group and the healthy group in PC1, as shown in Fig. 2C.

## 4 Discussion

In this study, we introduced a novel screening method that combines cancer‐specific tumor markers and glycan alterations for the early detection of PAC and HCC. Our findings indicated that α1‐antitrypsin with a fully sialylated biantennary glycan liked to asparagine 271 (AT271‐FSG) and α2‐macroglobulin with a fully sialylated biantennary glycan linked to asparagine 70 (MG70‐FSG) effectively distinguished patients with cancers from healthy controls. Previous studies have reported that α1‐antitrypsin [41] and α2‐macroglobulin [42] undergo glycosylation changes during cancer progression. However, our study uniquely highlights that these glycosylation alterations occur at specific sites on these proteins specifically during the early stages of cancers. Additionally, we achieved ROC‐AUC scores of up to 0.996 by using machine learning to integrate data from nine tumor markers, two glycopeptides, and 1688 EGPs, demonstrating superior diagnostic accuracy. The CSGSA score proved effective in detecting early stage cancers (stages I and II); however, its weak correlation with disease progression suggests it may relate more to cancer onset, making it a valuable tool for surveillance. Furthermore, the prevalence‐corrected PPVs obtained using CSGSA were 34.7% for PAC and 27.9% for HCC, which are significantly higher than industry benchmarks, where reported PPVs are usually below 1% [43]. While cancers with lower prevalence generally have lower PPVs, our method achieved PPVs over 30%, indicating its strong potential as a reliable, noninvasive diagnostic tool for practical screening. Compared to other emerging technologies like liquid biopsy [44] and genetic sequencing [45, 46], CSGSA offers notable advantages in accuracy, cost‐effectiveness, and reproducibility, highlighting its potential for broader practical applications in cancer diagnostics.

However, this study has several limitations. (i) The retrospective nature of the study limits the ability to confirm the efficacy of CSGSA, and a prospective approach is needed to determine if it can effectively identify cancers in asymptomatic individuals [47]. (ii) It is essential to distinguish liver and pancreatic cancers from other types, such as lung, colorectal, prostate, or gastric cancers, to enhance selectivity. (iii) The number of specimens used to develop the machine learning model was limited, particularly for HCC, which may have contributed to the lower predictive accuracy observed in this group. While our cross‐validation approach involves each sample being included in the training set across different iterations, the training and validation sets within each individual run were strictly separated and mutually exclusive. Thus, we consider the risk of data leakage to be negligible. Nevertheless, the combination of a relatively small sample size and high‐dimensional input features (EGPs) may increase the potential for overfitting through other mechanisms. In such cases, strong classification performance observed in the training data may not necessarily translate to equally high accuracy in the validation set. To ensure the generalizability and robustness of the model in real‐world applications, future studies incorporating independent external cohorts for validation will be essential. (iv) While this study used AFP as a liver cancer marker, it did not include PIVKA‐II, a tumor marker for diagnosing HCC that is produced in cases of vitamin K deficiency or inhibition. PIVKA‐II is notable for its ability to detect HCC at an earlier stage and with greater specificity than AFP. Many patients in this study had abnormal values caused by vitamin K deficiency from warfarin use or cirrhosis, leading to the exclusion of PIVKA‐II from the analysis. However, including PIVKA‐II in the diagnostic model could enhance the accuracy of HCC detection. (v) This study did not completely identify the glycopeptides that distinguish patients with chancers from healthy individuals. Unlike proteomics, which focuses solely on identifying protein species, glycoproteomics encompasses both protein identification and the determination of glycosylation sites and glycan structures. Additionally, while proteomics benefits from extensive MS/MS libraries and databases, similar resources for glycopeptides are lacking due to the complexity of their MS/MS patterns. Although we have successfully identified several glycopeptide features as cancer markers in previous studies [26, 27, 28], these efforts were time‐consuming and labor‐intensive. Given these challenges, we chose not to identify all 1688 glycopeptides in this study, opting instead to increase throughput and focus on processing a large number of samples. However, we expected that continuous updates to the glycopeptide database and advancements in mass spectrometry sensitivity will enable the identification of more glycopeptides. Another limitation involves the molecular weight cutoff used during glycopeptide enrichment. Although we applied a 10 kDa filter, its effective cutoff is not absolute, and in practice, glycopeptides larger than 4 kDa are preferentially retained, while smaller peptides often pass through. This may result in the loss of smaller glycopeptides or retention of some large nonspecific peptides. However, based on our previous work, this method effectively enriches a wide range of glycopeptides while excluding the majority of nonglycosylated peptides, offering a practical balance between specificity and throughput for biomarker discovery.

Accurately identifying cancer patients is crucial, but reducing false positives is also critical, particularly in screenings where only a few cases are detected among a large healthy population. A false‐positive rate above 5% can significantly lower the PPV to below 1% for many cancers, making the test impractical for clinical use. A PPV under 1%, even with high sensitivity, is unsuitable for screening due to the unnecessary stress on patients and the healthcare system. The high PPV of over 10% achieved by our screening method suggest its potential as a first‐line, noninvasive test in routine clinical practice, potentially decreasing the need for more invasive procedures like ultrasound examination. Implementing this screening approach could therefore not only improve patient outcomes but also significantly reduce healthcare costs associated with late‐stage cancer treatments. Our study also highlights the potential of integrating advanced machine learning techniques with molecular diagnostics. Future research could leverage newer machine learning models to further enhance the sensitivity and specificity of the diagnostic process. Additionally, ongoing updates to the glycopeptide databases and advancements in mass spectrometry sensitivity could yield even more robust datasets for analysis.

## 5 Conclusions

Comprehensive serum glycopeptide spectra analysis (CSGSA) using neural network model (Model 3) successfully discriminated PAC and HCC from healthy controls, with ROC–AUCs of 0.997 and 0.995, respectively, outperforming existing tumor markers. It also identified Stage I and II cases with ROC‐AUCs of 0.999 (PAC) and 0.997 (HCC), enabling early stage detection. The PPVs have reached 34.7% for PAC, and 27.9% for HCC, making this approach practically applicable. This approach holds promise for enhancing cancer diagnosis by alerting asymptomatic individuals to the onset of liver and pancreatic cancers.

## Conflict of interest

LSI Medience Corporation will offer cancer screening services. LSI Medience Corporation has applied for a patent related to this research in Japan (Toku‐gan 2023‐105968). The other authors declare that they have no competing interests.

## Author contributions

MK, TK, and KT conceptualized and designed the study. MK and KT reviewed previous research and formulated the hypothesis. YT and KK were in charge of recruiting participants, obtaining written informed consent, preparing specimens, collecting clinical data, and creating case report forms. KT developed a machine learning model using Python. MM established the basic concepts involved in CSGSA using ovarian cancer specimens. CH performed glycopeptide analysis using LC–MS and was responsible for identifying putative glycopeptide features, as well as validating the methodology. MK and KT wrote the original draft. TK supervised the study. All authors reviewed and edited the final manuscript and made the decision to submit it.

## Supporting information


**Appendix S1.** 1688 EPGs information: m/z and retention time.

**Fig. S1.** Principal component analysis of the training, validation, and hold‐out test sets.
**Table S1.** Demographic characteristics of the patients: Training and validation sets.
**Table S2.** Demographic characteristics of the patients: Hold‐out test set.

## Data Availability

The levels of nine tumor markers, AT271‐FSG, MG70‐FSG, and EGPs expression data, along with anonymized case information, will be available for disclosure after publication. Researchers interested in accessing the raw data should contact the corresponding authors via e‐mail. Access will require approval from each institution's ethics committee and a collaborative agreement with the corresponding authors.
