# Supplementary material for: Machine learning for identifying liver and pancreas cancers through comprehensive serum glycopeptide spectra analysis: a case‐control study
Source: Mol Oncol. 2025 Jun 30;19(12):3499–517. doi: 10.1002/1878-0261.70084 (PMC12688161; doi:10.1002/1878-0261.70084)
Supplement: Supplementary file 2 — Fig. S1. Principal component analysis of the training, validation, and hold‐out test sets. Table S1. Demographic characteristics of the patients: Training and validation sets. Table S2. Demographic characteristics of the patients: Hold‐out test set. [file MOL2-19-3499-s001.pdf]

Machine learning for identifying liver and pancreas cancers through comprehensive serum glycopeptide spectra analysis: a case control study

Motoyuki Kohjima[1], Yuko Takami[2], Ken Kawabe[1], Kazuhiro Tanabe[3], Chihiro Hayashi[3], Mikio Mikami[4], Tetsuya Kusumoto[5]

[1]Department of Gastroenterology, NHO Kyushu Medical Center, Fukuoka, Japan.

[2]Department of Hepato-Biliary-Pancreatic Surgery, NHO Kyushu Medical Center, Fukuoka, Japan.

[3]Medical Solution Promotion Department, Medical Solution Segment, LSI Medience Corporation, Tokyo, Japan

[4]Chigasaki Central Hospital, Women's Center, Kanagawa, Japan

[5]Department of Gastrointestinal Surgery and Clinical Research Institute Cancer, Research Division, NHO Kyushu Medical Center, Fukuoka, Japan.

Supporting Tables

Table S1. Demographic characteristics of the training and validation sets

Condition	Age	Number	Sex (Man Ratio)	Stage	Race
Healthy Volunteers (HE)	47.6 (±12.4)	540	50.7%		Asian (247)<br>Caucasian (114)<br>African American (115)<br>Hispanic (63)<br>Mixed Ethnicities (1)
Pancreatic Cancer (PAC)	66.1 (±8.6)	105	43.7%	Stage I (16)<br>Stage II (43)<br>Stage III (9)<br>Stage IV (31)<br>Unclassified (6)	Asian (41)<br>Caucasian (64)
Hepatocellular Carcinoma (HCC)	71.3 (±9.3)	41	63.4%	Stage I (2)<br>Stage II (27)<br>Stage III (11)<br>Stage IV (1)	Asian (41)
Total	51.8 (±14.3)	686	50.4%		

The numbers in the parentheses indicate the standard deviation of the age or number of participants.

Table S2. Demographic characteristics of the hold-out test set

Condition	Age	Number	Sex (Man Ratio)	Stage	Race
Healthy Volunteers (HE)	55.0 (±8.5)	50	46.0%		Asian (50)
Pancreatic Cancer (PAC)	70.0 (±7.6)	14	42.9%	Stage I (1)<br>Stage II (1)<br>Stage III (8)<br>Stage IV (4)	Caucasian (14)
Hepatocellular Carcinoma (HCC)	69.0 (±9.0)	8	87.5%	Stage II (0)<br>Stage II (2)<br>Stage III (1)<br>Stage IV (1)<br>Unclassified (4)	Asian (8)
Total	59.5 (±10.7)	72	50.0%		

The numbers in the parentheses indicate the standard deviation of the age or number of participants.

Supporting Figure

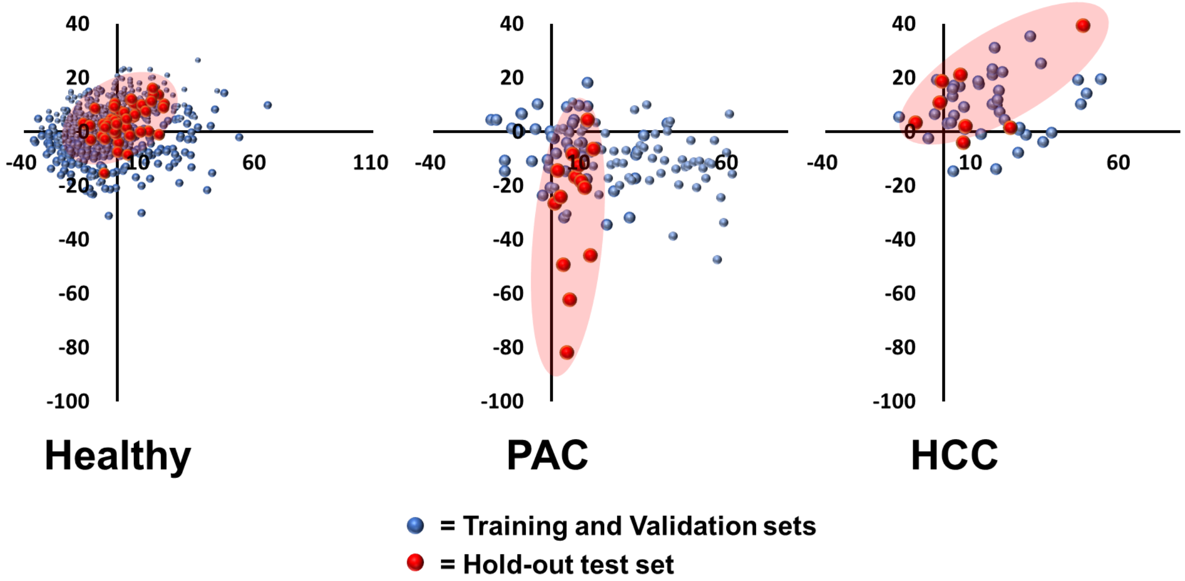

Fig. S1. Principal component analysis of the training, validation, and hold-out test sets. Score plots derived from principal component analysis (PCA) are shown, comparing the distributions of the training/validation sets (dark blue) and the hold-out test set (red).
